# Primary Hyperoxaluria Type 1 Disease Manifestations and Healthcare Utilization: A Multi-Country, Online, Chart Review Study

**DOI:** 10.3389/fmed.2021.703305

**Published:** 2021-09-20

**Authors:** Xiangling Wang, David Danese, Thomas Brown, Jessica Baldwin, Gautam Sajeev, Erin E. Cook, Yao Wang, Chunyi Xu, Hongbo Yang, Michael L. Moritz

**Affiliations:** ^1^Center for Personalized Genetic Healthcare, Department of Nephrology and Hypertension, Department of Molecular Medicine, Cleveland Clinic, Cleveland, OH, United States; ^2^Alnylam Pharmaceuticals, Inc., Cambridge, MA, United States; ^3^Analysis Group, Inc., Boston, MA, United States; ^4^Division of Nephrology, Department of Pediatrics, University of Pittsburgh School of Medicine, Pittsburgh, PA, United States

**Keywords:** primary hyperoxaluria type 1, oxaluria, chart review, healthcare resource utilization, kidney stones, chronic kidney disease, urinary tract infections

## Abstract

**Background:** Primary hyperoxaluria type 1 (PH1) is a rare genetic disease that can result in irreversible damage to the kidneys and, eventually, extrarenal organs. While kidney failure is a known consequence of PH1, few studies to date have characterized clinical consequences of PH1 prior to kidney failure, and data on healthcare resource use outcomes across different stages of disease severity in PH1 are also limited. To help fill this knowledge gap, this study characterized the clinical and healthcare resource use (HRU) burden in patients with PH1 with varying stages of kidney disease.

**Methods:** Nephrologists in the United States, Canada, United Kingdom, France, Germany, and Italy abstracted chart data from patients with PH1 under their care via an online questionnaire. Eligible patients had confirmed PH1 and ≥2 office visits from 2016 to 2019.

**Results:** A total of 120 patients were analyzed (median age at diagnosis, 17.4 years old, median age at index 19.5 years old, median eGFR at index 45 ml/min/1.73 m^2^; median follow-up 1.7 years). During follow-up, the most common PH1 manifestations were kidney stones and urinary tract infections (UTIs, both 56.8%), and the most common symptoms were fatigue/weakness (71.7%) and pain (64.6%). With regard to HRU during follow-up, 37.4% required lithotripsy, 31.3% required ureteroscopy, and 9.6% required nephrolithotomy. PH1-related hospitalizations and emergency/urgent care visits were noted for 84.0 and 81.6% of patients, respectively.

**Conclusions:** The current study demonstrated that patients with PH1 across various stages of kidney disease exhibited a substantial clinical burden, including kidney stones, UTIs, fatigue/weakness, and pain, and required frequent HRU, including kidney stone procedures, hospitalizations, and emergency visits. These findings highlight the significant morbidity and HRU burden in patients with PH1.

## Introduction

Primary hyperoxaluria type 1 (PH1) is a rare, autosomal recessive genetic disease resulting from mutations in the *AGXT* gene. PH1 causes irreparable damage to the kidneys and other vital organs, with potentially life-threatening consequences. The estimated prevalence of PH1 is 1–3 per 1,000,000 in Europe and North America, with greater prevalence in populations with higher levels of consanguinity (e.g., Middle Eastern and North African populations) or where founder mutations are present (e.g., Canary Islander populations) (1–6). While progression to kidney failure has been well-characterized in PH1 natural history studies, a better understanding of the disease burden prior to kidney failure and the healthcare resource use (HRU) outcomes experienced across all stages of PH1 will be important for development of efficient treatments.

In patients with PH1, *AGXT* gene mutations lead to deficiency in the alanine-glyoxylate aminotransferase (AGT) enzyme, which catalyzes the conversion of glyoxylate to glycine in the liver (7). As a result, glyoxylate accumulates and, rather than being primarily metabolized to glycine, is converted to oxalate, which is then excreted by the kidneys (7). In the kidneys, the excess oxalate combines with calcium to form insoluble calcium oxalate (CaOx) crystals, which can result in kidney stones, nephrocalcinosis and progressive kidney damage, ultimately leading to kidney failure (4, 7, 8). The eventual loss of kidney function and the inability of kidneys to fully clear oxalate from blood can result in systemic oxalosis, in which oxalate accumulates in plasma and leads to extrarenal CaOx crystal formation, with resultant damage to organs such as bone, heart, skin, and eyes (9, 10). In addition to carrying a significant clinical burden, PH1 also has important humanistic impacts. A recent survey found that manifestations of PH1 and disease management measures often create challenges for patients in terms of pain and other physical trauma while also disrupting the lives of patients and their caregivers (11). The same survey found that a majority of patients and caregivers experienced ongoing emotional stress due to uncertainty over when a painful stone event, complication from oxalosis, or kidney failure might occur.

Common supportive care measures, including hyperhydration and crystallization inhibitors, are effective to increase oxalate solubility and prevent CaOx crystallization but do not target the underlying pathophysiology of PH1 (4, 12, 13). Pyridoxine may stabilize certain pathogenic AGT variants to enhance AGT enzyme activity, with some degree of efficacy in~30% of patients (9). Later-stage management consists of intensive dialysis as a bridge to transplant or when no other viable treatment option exists (13–15). Liver transplantation, by addressing the metabolic defect in PH1, can normalize oxalate levels and, if done preemptively, prevent progression to kidney failure; however, it carries substantial morbidity and mortality risk and must be paired with kidney transplantation to restore lost kidney function in patients with kidney failure. To address the limitations of current options, multiple novel PH1 therapies are in clinical testing, including RNA interference (RNAi) therapeutics that suppress expression of proteins involved in oxalate production and an enteric oxalate-degrading bacterial preparation (16–18).

To date, literature on the natural history of PH1 has typically focused on the outcomes of kidney failure and mortality. Data on other outcomes, including clinical outcomes occurring prior to kidney failure and HRU outcomes in PH1, are less common. To complement the existing evidence base, a multi-country, retrospective, online chart review study was conducted to characterize the clinical and HRU burden of PH1 at various stages of kidney disease, with a focus on disease burden prior to kidney failure.

## Materials and Methods

### Study Overview

A retrospective chart review study of patients with PH1 under nephrologists' care in the United States (US), United Kingdom (UK), France, Germany, Italy, and Canada was conducted. Participating nephrologists were asked to abstract chart data for randomly selected patients who met the study eligibility criteria and enter this data into a standardized online questionnaire prepared by the investigators. The online questionnaire was pilot tested for clarity and usability by 2 US-based nephrologists with experience managing PH1. The form was revised based on their input and translated into French, German, and Italian. All patient data collected were anonymous and non-identifiable. Physicians completed data entry between October 2019 and January 2020. The study was approved by the New England Independent Review Board in October 2019.

### Data Source and Physician Eligibility

Adult and pediatric nephrologists were recruited from the Schlesinger Group healthcare and partner panels for this study. These online panels include over 125,000 physicians whose credentials, including demographics and education, are verified by Schlesinger Group. Panel members are preregistered to be contacted for opportunities to participate in surveys and chart review studies. An invitation link for the current study was sent to adult and pediatric nephrologists in the panel. In addition, following the initial invitation, panel participants were contacted periodically with follow-up reminders to encourage participation and maximize response rates. Respondents to the survey link were asked to first complete screening questions to determine their eligibility to participate in this chart review study (see Supplementary Figure 1, Part I: Physician Practice Characteristics). To allow collection of data characterizing a contemporary cohort of patients with PH1, physicians were required to meet the following criteria to be eligible to participate: (1) he/she was a practicing adult or pediatric nephrologist; (2) he/she was the healthcare provider primarily responsible for managing ≥1 patient with PH1 during the past 3 years; and (3) he/she had ≥1 patient with PH1 who met the patient selection criteria summarized below. Physicians were excluded if they did not complete the eligibility questions, did not meet these study eligibility criteria, or if they met the criteria but did not provide data for any eligible patients. As compensation for their time and effort, physicians received an honorarium of $95–155 (USD) for each patient chart provided.

### Patient Selection

Participating physicians reviewed the patient selection criteria to determine the eligibility of their patients. To be eligible, patients had to have: a PH1 diagnosis confirmed by genetic test or liver biopsy-based AGT assay; ≥2 office visits to the participating physician in the last 3 years; and available medical charts with information on signs, symptoms, disease manifestations, and PH1-related HRU since their first visit to the participating physician in the last 3 years. If a physician indicated that they had more than one eligible PH1 patient under their care, they were asked to randomly select patients for inclusion in this study, one at a time, up to a maximum of 5 patients. To facilitate random selection of each patient, the online questionnaire interface displayed a random letter of the alphabet, and physicians were instructed to select the patient whose surname started with the letter closest to that randomly generated letter.

### Study Period

The index date was defined as the date of patients' first office visit in the past 3 years prior to chart abstraction. Restriction of data collection to the past 3 years was done to yield an approximate snapshot of the current burden of prevalent PH1 cases. The baseline period was defined as the time prior to the index date and the follow-up period defined as the time from the index date to the most recent office visit prior to chart abstraction.

### Study Measures

All data were collected via the standardized online questionnaire, which included questions about the participating physician and chart data for each patient (Supplementary Figure 1). Data collected on participating physicians included specialty, years in practice, practice setting, and number of patients with PH1 seen in the last 3 years. Data collected on patients included demographics at index, diagnostic information, and clinical, HRU, and disease management history prior to index and during the follow-up period. Unless otherwise noted in the questionnaire, clinical and HRU outcomes were defined pragmatically, as recorded by physicians in patient charts based on their interpretation of the available data (rather than by an external “gold standard” definition based on laboratory criteria, imaging, and/or any other criteria specified in the online questionnaire). After starting data entry, physicians could pause and return to the questionnaire as needed, with no time limit for completion. Data were included for all eligible patients for whom the physician reached the end of the questionnaire and confirmed completion via the online platform (with no required minimum number of items to complete).

### Statistical Analyses

Descriptive analyses of physician and patient data were performed. For each variable, these analyses included physicians/patients with available data on that variable (with the number of physicians/patients with available data for each analysis reported as ‘*N*’ in the Results). Categorical variables were summarized with counts and percentages and continuous variables summarized with means, standard deviations (SDs), medians, and interquartile ranges (IQRs). Rates of acute clinical events and HRU during follow-up were calculated as the total number of events per person-year (PY).

## Results

### Physician Characteristics

**Figure 1:**
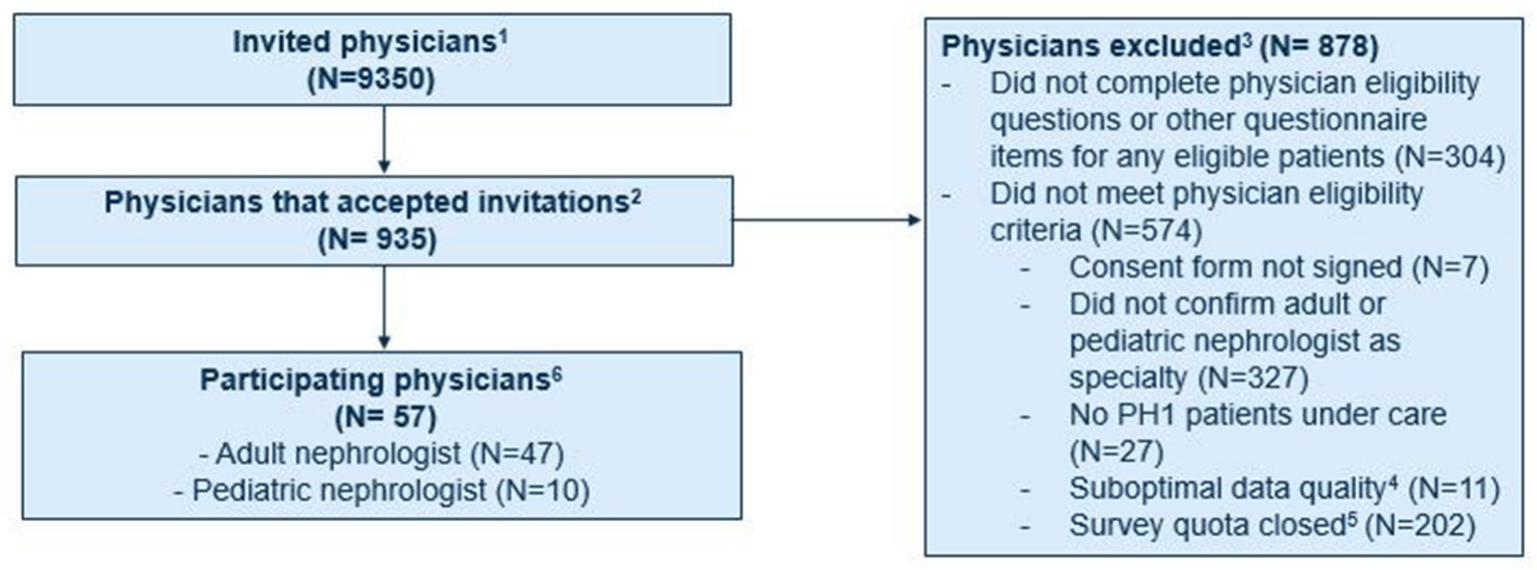
Recruitment of physicians participating in chart review study. ^1^Adult and pediatric nephrologists who were part of the Schlesinger Group healthcare and partner panels who were notified of the opportunity to participate in this study. ^2^Physicians who accepted the invitation and were asked to complete screening questions to determine their eligibility to participate in the study. ^3^Physicians who did not complete the eligibility criteria, did not provide data for any eligible patients, and did not meet the study eligibility criteria.^4^Included providers who selected responses that were illogical (e.g., reported number of patients under their care was larger than possible based on responses to initial screening questions) and were thus excluded from the study. ^5^Includes providers who accepted invitation after quotas for their respective country and/or specialty (as implemented during physician recruitment to ensure representation of patients from different countries and age groups) had been met. ^6^Physicians who met the study eligibility criteria and provided information on 120 eligible patients with PH1.

The study invitation was sent to approximately 9,350 physicians and accepted by 935 (Figure 1). Of these, 609 did not meet the eligibility criteria and 269 did not complete the questionnaire. The remaining 57 physicians met the eligibility criteria and provided information on 120 patients with PH1. Approximately one-third were based in the US (19 physicians/37 patients), with smaller proportions in France (10/19), Italy (8/15), the UK (8/18), Canada (7/16), and Germany (5/15). All participating physicians were nephrologists, including 17.5% who were pediatric nephrologists. These physicians had been practicing in their field for a mean of 16.8 years (SD: 7.4), had a median of 6 patients (IQR: 3, 23) with PH1 under their care with ≥2 office visits in the last 3 years, and provided data on a mean of 2.11 eligible patients under their care. Similar percentages of participating physicians were practicing at academic (53%) and non-academic institutions (47%).

### Patient Characteristics at PH1 Diagnosis

Median patient age at diagnosis (*N* = 82) was 17.4 years (IQR: 11.1, 24.9). The majority of patients were white (78.2%) and male (64.4%). Of those with available eGFR data at diagnosis (*N* = 86), 15.1% had eGFR ≥ 90 ml/min/1.73 m^2^, 33.7% had eGFR 60–89, 41.9% had eGFR 30–59, 7.0% had eGFR 15–29, and 2.3% had eGFR < 15. Most patients (82.0%) presented with signs and/or symptoms of PH1, with a median age of 13.0 years at symptom onset (*N* = 111). The most common signs and symptoms at diagnosis (*N* = 111) were kidney stones (51.4%), fatigue/weakness (44.1%), and pain (44.1%). Mean and median times from symptom onset to diagnosis were 5.7 (SD: 8.3) and 2.8 years (IQR: 0.9, 6.7), respectively (*N* = 82).

### Patient, Disease, and Treatment Characteristics at Index

Median patient age at index (*N* = 99) was 19.5 years, with 58.6% of patients ≥18 years old (Table 1). Median duration of post-index follow-up was 1.7 years. Of those with genetic data available (*N* = 87), 9.2% had two p.Gly170Arg pathogenic variant *AGXT* alleles, 21.8% had one p.Gly170Arg *AGXT* allele, and the remainder had two pathogenic variant *AGXT* alleles not encoding the p.Gly170Arg amino acid substitution. The remaining patients included in the cohort had their PH1 diagnosed via biopsy-based assessment of hepatic AGT enzyme activity. Among patients with available data (*N* = 81), median eGFR at index was 45 ml/min/1.73 m^2^ (IQR: 35, 75); 37.1% of patients had eGFR ≥ 60 ml/min/1.73 m^2^ at index. With regard to PH1 management history (*N* = 111), hyperhydration (70.3%), and crystallization inhibitor use (60.4%) were reported in most patients; 40.5% of patients had a history of pyridoxine use.

**Table 1:** Baseline patient demographics and clinical characteristics.

	**Value**
**Demographics (n contributing data)**	
Age group at index visit (*N* = 99)	
*n* (%) 0–<2 years	3 (3.0%)
*n* (%) 2–<6 years	2 (2.0%)
*n* (%) 6–<18 years	36 (36.4%)
*n* (%) ≥18 years	58 (58.6%)
Age at index, years (*N* = 99)	
Mean ± SD	22.4 ± 12.5
Median (IQR)	19.5 (15.7, 28.7)
Gender (*N* = 118)	
*n* (%) female	42 (35.6%)
*n* (%) male	76 (64.4%)
Race (*N* = 119)	
*n* (%) White or Caucasian	93 (78.2%)
*n* (%) Black	12 (10.1%)
*n* (%) Asian	10 (8.4%)
*n* (%) Other	4 (3.4%)
**Clinical characteristics (*n* contributing data)**	
Diagnosis relative to index date (*N* = 94)	
*n* (%) diagnosed before index	62 (66.0%)
*n* (%) diagnosed on or after index	32 (34.0%)
Time from diagnosis to index in years (*N* = 62)	
Mean ± SD	3.7 ± 4.9
Median (IQR)	1.1 (0.20, 5.9)
eGFR at index (*N* = 81)	
*n* (%) eGFR ≥ 90 mL/min/1.73m^2^	11 (13.6%)
*n* (%) eGFR 60–89 mL/min/1.73m^2^	19 (23.5%)
*n* (%) eGFR 30–59 mL/min/1.73m^2^	34 (42.0%)
*n* (%) eGFR 15–29 mL/min/1.73m^2^	10 (12.3%)
*n* (%) eGFR <15 mL/min/1.73m^2^	7 (8.6%)
Mean ± SD	52.9 ± 29.4
Median (IQR)	45.0 (35.0, 75.0)
Treatment history (past or current) at index (*N* = 111)	
*n* (%) hyperhydration	78 (70.3%)
*n* (%) crystallization inhibitors	67 (60.4%)
*n* (%) pyridoxine	45 (40.5%)
*AGXT* pathogenic variant status (*N* = 87)	
*n* (%) with 0 p.Gly170Arg alleles	60 (69.0%)
*n* (%) with 1 p.Gly170Arg allele	19 (21.8%)
*n* (%) with 2 p.Gly170Arg alleles	8 (9.2%)

*eGFR, estimated glomerular filtration rate; IQR, interquartile range; N, number; SD, standard deviation. Note: Index date was defined as the first visit date within the last 3 years*.

### Disease Manifestations During Follow-Up

During the follow-up period, the most common signs and symptoms (*N* = 113–114) of PH1 were fatigue/weakness (71.7%), pain (64.6%), and hematuria (defined per data collection form as “blood in urine”: 57.9%, Table 2). Common clinical events (*N* = 118) included kidney stone events and urinary tract infections (UTIs; each reported ≥ 1 time in 56.8%). In the overall population, kidney stone events occurred at a rate of 0.8 per PY and UTIs at a rate of 1.0 per PY (Supplementary Figure 2).

**Table 2:** Prevalence of clinical signs, symptoms, and other PH1 manifestations during follow-up.

**PH1 manifestation (*n* contributing data)**	***n*** **(%) with manifestation**
Clinical signs (*N* = 114)	
Hematuria	66 (57.9%)
Decreased urine output	44 (38.6%)
Other	12 (10.5%)
None of the above	16 (14.0%)
Symptoms (*N* = 113)	
Fatigue/weakness	81 (71.7%)
Pain	73 (64.6%)
Other	11 (9.7%)
None of the above	13 (11.5%)
Other manifestations (*N* = 118)	
Stone episodes	67 (56.8%)
UTI/pyelonephritis	67 (56.8%)
Nephrocalcinosis	33 (28.0%)
Acute kidney decline	32 (27.1%)
Other	6 (5.1%)
None of the above	17 (14.4%)

*N, number; UTI, urinary tract infection. Note: Hematuria was defined as “blood in urine.” Acute kidney decline was defined as an acute eGFR decline by 20% or more*.

With regard to kidney function, 32 patients (27.1%) had an episode of acute decline (*N* = 118); among the 27 of these 32 patients for whom more detailed information was provided, common causes of acute decline included dehydration, infection, and obstructive stone events (Supplementary Figure 3). Of the 27 patients in question, 18 (66.7%) experienced a permanent loss of kidney function (defined as failure to recover to the level of kidney function seen immediately prior to the episode) in association with an episode of acute decline. About one fifth of patients (20.4%) in the overall cohort (n/*N* = 23/113) ever had kidney failure (captured as “end-stage renal disease” in the data collection form), which was diagnosed at a median age of 25.3 years (*N* = 16, Table 3), and approximately one-fifth (21.4%, *n*/*N* = 24/112) of patients required dialysis (before or during follow-up), with a median age of 24.6 years at the time of initiation (*N* = 16). In addition, transplant (before or during follow-up) was performed for 15.9% of patients (*n*/*N* = 18/113), of whom 61.1% had combined liver-kidney transplant.

**Table 3:** Late-stage outcomes of PH1.

**Late-stage outcomes (*n* contributing data)**	**Value**
**Dialysis**	
Patients who ever received dialysis (*N* = 112)	
*n* (%) yes	24 (21.4%)
*n* (%) no	88 (78.6%)
Most recent dialysis type (*N* = 24)	
*n* (%) hemodialysis	22 (91.7%)
*n* (%) peritoneal dialysis	2 (8.3%)
Age at dialysis initiation in years (*N* = 16)	
Mean ± SD	28.1 ± 11.8
Median (IQR)	24.6 (20.3, 34.5)
Time from diagnosis to initiation of dialysis in years (*N* = 12)	
Mean ± SD	4.0 ± 5.1
Median (IQR)	2.5 (0.5, 5.3)
Kidney failure^*^	
Patients who ever had kidney failure (eGFR < 15 ml/min/1.73 m^2^) (*N* = 113)	
*n* (%) yes	23 (20.4%)
*n* (%) no	90 (79.7%)
Age at diagnosis of kidney failure, years (*N* = 16)	
Mean ± SD	27.7 ± 11.9
Median (IQR)	25.3 (20.5, 34.2)
Diagnosis of kidney failure relative to PH1 diagnosis (*N* = 23)	
*n* (%) diagnosis of kidney failure at or after PH1 diagnosis	11 (47.8%)
*n* (%) diagnosis of kidney failure before PH1 diagnosis	3 (13.0%)
*n* (%) unknown	9 (39.1%)
Time from PH1 diagnosis to kidney failure in years (*N* = 11)	
Mean ± SD	3.6 ± 5.6
Median (IQR)	1.9 (0.2, 3.4)
**Transplants**	
Patients who ever underwent transplant (*N* = 113)	
*n* (%) yes	18 (15.9%)
*n* (%) no	95 (84.1%)
Type of transplant among patients with transplant (*N* = 18)	
*n* (%) isolated kidney transplant	4 (22.2%)
*n* (%) isolated liver transplant	6 (33.3%)
*n* (%) combined liver-kidney transplant	11 (61.1%)
Time from diagnosis to first transplant in years (*N* = 10)	
Mean ± SD	5.8 ± 7.6
Median (IQR)	2.0 (1.0, 8.1)
Time from dialysis initiation to first transplant in years (*N* = 7)	
Mean ± SD	4.2 ± 4.8
Median (IQR)	2.7 (1.0, 5.2)
**Vital status**	
Vital status on survey date (*N* = 110)	
*n* (%) death	9 (8.2%)
Age at death in years (*N* = 5)	
Mean ± SD	27.8 ± 17.6
Median (IQR)	35.0 (21.0, 35.0)

*IQR, interquartile range; N, number; SD, standard deviation.*

* *Captured as “end-stage renal disease” (defined as being in CKD stage 5 with need for dialysis) in the online chart data abstraction form. Note: Dialysis may be initiated prior to kidney failure in patients with PH1. The number of patients with transplants adds to more than 18 because some patients had multiple types of transplants*.

Aside from renal manifestations, systemic manifestations of PH1 (*n*/*N* = 36/104) were reported in 34.6% of patients during the follow-up period—most often anemia, arrhythmias, and bone pain (Supplementary Figure 4).

### HRU During Follow-Up

During the follow-up period, 84.0% of patients required hospitalization (*N* = 94) for reasons related to PH1 and 81.6% required ≥1 emergency visit (*N* = 87, Table 4) related to PH1. PH1-related hospitalizations and emergency visits each occurred at a rate of 1.1 per PY, and median length of stay was 5.0 days (IQR: 3.0, 7.5).

**Table 4:** PH1-related hospitalizations, urgent/emergency care visits, and outpatient visits during follow-up.

**Visit type (*n* contributing data)**	**Value**
Hospitalizations (*N* = 94)	
*n* (%) of patients with ≥ 1 during follow-up	79 (84.0%)
Total number during follow-up	167
Total follow-up among patients with available data	159.2 patient-years
Rate	1.1 per patient-year
Length of stay, mean ± SD	6.8 ± 11.5 days
Length of stay, median (IQR)	5.0 (3.0, 7.5)
Urgent or emergency care visits (*N* = 87)	
*n* (%) of patients with ≥ 1 during follow-up	71 (81.6%)
Total number during follow-up	161
Total follow-up among patients with available data	140.7 patient-years
Rate	1.1 per patient-year
Outpatient visits (*N* = 89)	
*n* (%) of patients with ≥ 1 during follow-up	87 (97.8%)
Total number during follow-up	589
Total follow-up among patients with available data	150.4 patient-years
Rate	4.0 per patient-year

*IQR, interquartile range; N, number; SD, standard deviation*.

With regard to specific medical procedures (*N* = 115) related to PH1, approximately one-third of patients had ≥1 lithotripsy and approximately one-third had ≥1 ureteroscopy to manage kidney stones during follow-up (Table 5), while 9.6% had ≥1 percutaneous nephrolithotomy (PCNL). Patients with ≥1 lithotripsy required these procedures at a rate of 1.2 per PY; corresponding rates for patients with ≥1 ureteroscopy and ≥1 PCNL were 1.0 and 0.6 procedures per PY, respectively (Table 5). In addition, 4.3% of patients underwent gastrostomy tube (G-tube) placement and 6.1% underwent nasogastric tube (NG-tube) placement during follow-up.

**Table 5:** PH1-related medical procedures during follow-up.

**Procedure (*n* contributing data)**	**Value**
**Lithotripsy**	
*n* (%) of patients undergoing ≥ 1 lithotripsy (*N* = 115)	43 (37.4%)
Among patients with ≥ 1 lithotripsy and known number of procedures (*N* = 30)	
Total number performed	72
Total follow-up	59.2 patient-years
Rate	1.2 per patient-year
**Ureteroscopy**	
*n* (%) of patients undergoing ≥ 1 ureteroscopy (*N* = 115)	36 (31.3%)
Among patients with ≥1 ureteroscopy and known number of procedures (*N* = 30)	
Total number performed	51
Total follow-up	49.3 patient-years
Rate	1.0 per patient-year
**Percutaneous nephrolithotomy**	
*n* (%) of patients undergoing ≥ 1 PCNL (*N* = 115)	11 (9.6%)
Among patients with ≥ 1 PCNL and known number of procedures (*N* = 7)	
Total number performed	8
Total follow-up	12.7 patient-years
Rate	0.6 per patient-year

*PCNL, percutaneous nephrolithotomy*.

## Discussion

This multi-country, retrospective online chart review study characterizes a relatively large cohort of patients with PH1 in North America and Europe, particularly relevant given the rarity of PH1. Outside of large cohort studies involving the two leading PH registries (5, 19) and an additional study led by a French PH1 reference center (20), most other published reports on PH1 have involved <100 patients (6, 21–23). Prior published reports have provided limited data on clinical outcomes of PH1 before progression to kidney failure and mortality, and data on HRU in PH1 are scarce (6, 22, 24, 25). The current study aimed to better characterize the clinical and HRU burden of PH1 in patients at different stages of disease, most without kidney failure. In this study, many patients experienced adverse clinical events during the follow-up period, and over 80% had ≥1 hospitalization and/or emergency visit related to PH1. These findings highlight the significant clinical and HRU burden of PH1 even prior to kidney failure.

The clinical burden in this study was notable in terms of kidney stone events and UTIs, as in other published PH1 cohorts (6, 24, 26). In addition, pain, fatigue/weakness, and hematuria (defined as “blood in urine” for data collection purposes) were reported as common PH1 signs and symptoms in the current cohort. This clinical burden was accompanied by substantial HRU. Overall, 84% of patients required ≥1 hospitalization and 82% required ≥1 emergency visit related to PH1 during the follow-up period, with each occurring at a rate of approximately 1 per PY. To provide context, national data from the US, Europe, and Canada suggest hospitalization rates of 0.08–0.25 per PY and emergency visit rates of 0.08–0.48 per PY in the general population, despite its older age relative to the PH1 population (27–29).

Stone interventions were also common in the current study population, and patients who required lithotripsy, ureteroscopy, and PCNL to manage stones underwent such procedures on an annual to biannual basis, on average. In this study, clinical and HRU burden were present despite the typically moderate degree of kidney impairment and the finding that most patients were receiving supportive care for PH1, and despite the heterogeneous nature of the disease.

The current study helps further elucidate the clinical burden of PH1, particularly in terms of clinically evident stone events and other symptoms prior to development of kidney failure, and is among the first to collect detailed HRU data in PH1. Moreover, this study collected data from all (rather than only newly diagnosed) PH1 patients over a recent 3 year period. As a result, findings are more likely to reflect the burden of PH1 in the current, prevalent population.

For context, it is important to note that the current study cohort differed from previously reported PH1 cohorts in terms of age and degree of kidney disease at diagnosis. Patients in this study had a median age at diagnosis of 17.4 years, somewhat older than in other PH1 cohorts (7–17 years) (6, 30, 31). As most participating physicians were adult nephrologists, patients closer to the documented smaller, later peak in the bimodal distribution of age at PH1 diagnosis (23) may have been overrepresented in the current study. Additionally, age at diagnosis was unknown in this study for 32% of patients. Pediatric onset may have been more common in these patients, as dates of diagnosis may not have been accessible to physicians caring for adults who were diagnosed as children. Nonetheless, the median age at diagnosis in this study may make the findings less generalizable to patients diagnosed in infancy or early childhood.

With regard to kidney function, patients in the current study had varying levels of impairment at diagnosis, but overall, there were fewer patients with eGFR <15 mL/min/1.73m^2^ at diagnosis than in other published cohorts (6, 7, 24). The current study may have captured a milder population, given the comparatively high proportion of adult nephrologists involved and given that pediatric-onset PH1 tends to progress more rapidly. The findings of this study may thus be more generalizable to patients with PH1 diagnosed prior to kidney failure. As a result, they provide evidence of a substantial burden of PH1 even in patients with less severe kidney impairment than previously published cohorts.

Other methodological considerations should also be taken into account in interpreting these results. Due to differences among physicians in terms of chart data capture, some data elements were not consistently available. Despite an initial feasibility assessment of data availability, some data elements were less available than expected (e.g., eGFR at index, age at diagnosis). In addition, although pilot tests were conducted and the online questionnaire revised accordingly, the questionnaire may have been challenging to complete due to its comprehensive and detailed nature, which may have impacted data quality and completeness, particularly for clinical and HRU event counts. Also of note is that data on urine oxalate, a key indicator of PH1 disease activity, were not considered sufficiently complete for reporting and interpretation (unavailable within 31 days of index for 61% of patients). Analysis was further complicated by inter-laboratory variability in terms of urine oxalate assays and methods for calculating and reporting oxalate assay results. Aside from these considerations, the retrospective nature of the study itself introduces potential issues such as selection bias and non-random missingness of data.

An additional methodological consideration is that comprehensive data validation via review of source medical charts was not possible, given the panel-based design. To minimize the impact of this limitation, steps were taken to prospectively reduce the potential for invalid data entry—specifically, by adopting feedback from pilot testing (as previously noted) with nephrologists to optimize survey clarity and usability, and by incorporating automated logic checks throughout the survey to help ensure accurate data entry and flag potentially erroneous entries for review and revision as needed. In addition, post-survey queries on certain key data fields were made to selected participants whose responses for those fields were identified as being potentially invalid and required further clarification. Even in a scenario that would allow validation of source medical charts, it remains a limitation of chart review studies in general that the accuracy of chart documentation fundamentally limits data quality, as events documented in patient charts cannot be “re-observed” prospectively for the purposes of the study.

These considerations notwithstanding, the current work suggests several areas for additional investigation. For instance, further research in a population with younger age at presentation and diagnosis would be valuable to provide a more complete picture of the clinical and HRU burden of PH1, given the frequency with which PH1 presents in childhood. Selected findings from the current cohort also suggest previously underappreciated elements of clinical burden in PH1 that may warrant further exploration. For example, prior anecdotal reports and small case series have noted that progressive kidney damage in PH1 may be accelerated by acute declines in kidney function (e.g., due to obstruction of renal outflow by CaOx stones) (21, 24, 32). In the current study, acute kidney decline was reported in 27% of patients and was often triggered by common events that would be relatively benign in a non-PH1 setting (e.g., dehydration). Similarly, anemia and arrhythmia were reported in an appreciable portion of patients who experienced systemic manifestations of PH1 during follow-up. Both have been documented in PH1 (33–35) and are also known complications of CKD more broadly (36, 37); however, their prevalence in PH1 is not well characterized. While findings from the current study regarding acute declines in kidney function and systemic manifestations of PH1 are preliminary in nature, more in-depth investigation into these events could further elucidate their contribution to the disease burden of PH1.

Additional economic analyses also represent a valuable direction for future research in PH1. The ability to translate clinical and HRU data from the current study to PH1-related cost estimates is limited, given the multinational nature of the patient cohort (and the associated variation across countries in terms of healthcare systems and resource use costs) and the chart-based study design, as study designs based on healthcare reimbursement claims data are best-suited to generate quantitative cost estimates (38). Country-specific claims studies of this type may therefore be valuable for more precisely characterizing hospitalization-related and total costs specifically attributable to PH1.

In summary, the current study found that patients with PH1 experienced substantial clinical and HRU burden. Patients commonly experienced stone events and UTIs as well as symptoms such as pain, fatigue and weakness. This clinical burden was accompanied by an appreciable burden in terms of HRU. Most patients required hospitalizations and emergency visits for their PH1 (each annually, on average), and a number also required stone management interventions, which are known to carry risk of clinical complications and impair quality of life. The clinical and HRU burden of PH1 were evident despite the finding that most patients were being managed with standard supportive care measures and the moderate level of kidney impairment at index. In addition, this burden is superimposed on a background of progressive kidney decline that is well-documented in PH1. These findings highlight an ever-present risk of ongoing morbidity and significant HRU, which accompany the well-recognized, ongoing risk of progressive kidney decline in PH1, and underscore the urgency of effective PH1 treatment throughout the disease course.

## Data Availability Statement

The original contributions presented in the study are included in the article/Supplementary Material, further inquiries can be directed to the corresponding author.

## Ethics Statement

The studies involving human participants were reviewed and approved by the New England Independent Review Board. Written informed consent from the participants' legal guardian/next of kin was not required to participate in this study in accordance with the national legislation and the institutional requirements.

## Author Contributions

All authors participated in the study design, analysis and interpretation, manuscript composition and editing.

## Funding

This study was funded by Alnylam Pharmaceuticals, Inc.

## Conflict of Interest

DD and TB are employees of Alnylam. JB is a former employee of Alnylam; she is currently an employee of Vertex Pharmaceuticals. MM and XW are consultants of Alnylam. GS, EC, YW, CX, and HY are employees of Analysis Group Inc., which received funding for this research from Alnylam.

## Publisher's Note

All claims expressed in this article are solely those of the authors and do not necessarily represent those of their affiliated organizations, or those of the publisher, the editors and the reviewers. Any product that may be evaluated in this article, or claim that may be made by its manufacturer, is not guaranteed or endorsed by the publisher.
