# Supplementary material for: Primary Hyperoxaluria Type 1 Disease Manifestations and Healthcare Utilization: A Multi-Country, Online, Chart Review Study
Source: Front Med (Lausanne). 2021 Sep 20;8:703305. doi: 10.3389/fmed.2021.703305 (PMC8488346; doi:10.3389/fmed.2021.703305)
Supplement: Supplementary file 1 [file Data_Sheet_1.PDF]

Supplementary Material Table of Contents

Supplemental Figure 1. Data collection questionnaire 2

Supplemental Figure 2. Incidence rate of acute events associated with PH1 during follow-up 21

Supplemental Figure 3. Reasons for onset of acute kidney decline in PH1 22

Supplemental Figure 4. Prevalence of extrarenal manifestations of oxalosis during follow-up 23

Supplemental Figure 1. Data collection questionnaire

Dear Doctor,

Thank you for agreeing to participate in this study of patients with primary hyperoxaluria type 1 (PH1). PH1 is an ultra-rare, autosomal recessive, life-threatening disease that causes irreparable damage to the kidneys and other vital organs. Patients with PH1 are deficient in an enzyme that converts glyoxylate to glycine. Deficiency in this enzyme leads to the overproduction and accumulation of oxalate, which can lead to urolithiasis and nephrolithiasis, nephrocalcinosis, and ultimately chronic kidney disease (CKD), renal failure, systemic oxalosis, and death.

The main objective of this study is to identify patients with PH1 and describe their patient characteristics, clinical characteristics at diagnosis, resource utilization, clinical outcomes, and disease management characteristics.

You will be asked to:

- Provide summary information about your practice
- Identify patients meeting this study's eligibility criteria
- Extract information from charts/medical records of eligible patients, including patient demographics, PH1 clinical characteristics at diagnosis, PH1-related resource utilization outcomes, PH1 clinical outcomes, and disease management characteristics

It will take approximately 20 to 25 minutes to complete data entry for one patient. Please be assured that all information you provide will be kept confidential. No selling or solicitation will result from your participation in this survey.

[Show the following disclaimer to U.S. participants]

Disclaimer:
It is mutually understood and agreed that this Form is and shall remain compliant with the US HIPAA privacy regulations. By submitting this Form, you agree that all of your responses are HIPAA compliant and do not contain any individually identifiable health information.

[Show the following disclaimer to European participants]

Disclaimer:
It is mutually understood and agreed that this Form is and shall remain compliant with the GDPR privacy regulations. By submitting this Form, you agree that all of your responses are GDPR compliant and do not contain any individually identifiable health information. Please note that any dates that you enter will be randomly transposed to ensure compliance with GDPR privacy regulations.

Please confirm that you have read and understood the information presented above, and that you agree to participate in the study.

1= I agree
2= I do not agree **[TERMINATE]**

Part I – Physician Practice Characteristics

S1.

a. In what type of practice do you treat the majority of your patients?

1= Group practice
2= Solo practice
3= Other

b. Which of the following best describes the main practice setting in which you work?

1= Private practice
2= Academic institution
3= Non-academic public institution
4= Other

c. Which of the following best describes the location of your main practice setting?

1= Hospital based
2= Non-hospital based
3= Other

S2. **[Show S2 to US participants only]** Please indicate in which state you primarily practice. **[Dropdown with all states in the US]**

S3. Which of the following best describes your specialty?

1= Nephrologist (general)
2= Pediatric nephrologist
3= Other **[TERMINATE]**

S4. How many years have you been practicing as a [Insert S3]?
_____ year(s)

S5. Approximately how many patients under your care have a diagnosis of PH1 confirmed by a genetic test or a liver biopsy-based AGT assay?
_____ patient(s) **[If S5=0, TERMINATE]**

S6. Among the **[S5]** PH1 patients under your care, approximately how many patients had at least two office visits with you in the past 3 years between **[survey_date - 1096 days]** and **[survey_date]?**
_____ patient(s) **[If S6=0, TERMINATE]**

S7. Among the **[S6]** PH1 patients under your care who had at least two office visits between **[survey_date - 1096 days]** and **[survey_date]**, for approximately how many patients are you the doctor primarily responsible for managing PH1 (i.e. coordinating care and regularly monitoring your patients for symptoms, and disease manifestations)?
_____ patient(s) **[If S7=0, TERMINATE]**

S8. Among the **[S7]** PH1 patients under your care who had at least two office visits, approximately what proportion were less than 18 years old on the date of their first visit with you between **[survey_date - 1096 days]** and **[survey_date]?**

1 = < 50% of patients were < 18 years old
2 = ≥ 50% of patients are < 18 years old

Part II – Chart Eligibility Criteria and Random Patient Selection

To be eligible for chart/medical record abstraction, patients need to satisfy **ALL** of the inclusion criteria described below.

1. The patient needs to have their PH1 diagnosis confirmed by a genetic test or liver biopsy-based AGT assay

2. The patient needs to have at least two visits within the past 3 years between **[survey_date - 1096 days]** and **[survey_date]**

    a. The date of the patient's first PH1-related visit on or after **[survey date - 1096 days]** will be defined as the index date

3. The patient's chart/medical record needs to have detailed information available for the following after the index date:
    a. PH1-related healthcare resource use (e.g., number of hospitalizations and length of stay, number of outpatient visits)
    b. Signs, symptoms, and clinical manifestations of PH1

To be included in the chart/medical record review, alive and deceased patients can be considered as long as they meet the inclusion criteria listed above. Please use the following process to select eligible patients.

[If S7=1 show the following]
1. You indicated that you are the doctor primarily responsible for one patient who had a confirmed PH1 diagnosis and at least two office visits during the last 3 years. Please use the inclusion criteria above to assess the eligibility for this patient. If you believe this patient will be eligible, please click **next** when you have this patient's chart/medical record ready for data abstraction. Patient eligibility will be confirmed in the next section.

2. If you believe this patient is not eligible, please confirm below and exit the survey.

    1= I only have one patient's chart/medical record and believe this patient is not eligible for inclusion in this study and would like to exit the survey. **[Exit]**

[If S7 > 1 show the following]
1. You indicated that you are the doctor primarily responsible for multiple patients who have confirmed PH1 diagnoses and at least two office visits during the last 3 years. Considering these patients, please access the charts/medical records for the patient whose last name starts with or is closest to the letter **[Random letter].**

2. Please use the inclusion criteria above to assess this patient's eligibility. If you believe this patient will be eligible, please click **next** when you have this patient's chart/medical record ready for data abstraction. This will be the first patient you include for extraction. Patient eligibility will be confirmed in the next section.

3. If you believe this patient is not eligible, please click <u>here</u> to receive a new letter and start again with step 1 for another PH1 patient under your care.

4. If none of your PH1 patients meet the inclusion criteria, please confirm below and exit the survey.
   1= I don't have another patient's chart/medical record and would like to exit the survey. **[Exit]**

[If S7 > 1 show the following]
If the patient fails to meet one of the criteria, you can restart the random selection section to identify a new patient. If the patient meets all the inclusion criteria, you will be asked to continue to extract more detailed information from this patient's chart/medical record.

If you have more eligible patients whom you would like to provide information on, you will be able to return to this section after you complete data extraction for the currently selected patient. You can provide information for up to 5 eligible patients. Please answer all questions to the best of your ability.

Part III – Patient Selection Process

Now we will begin to collect information from the selected patient. The first set of questions will confirm the eligibility of the PH1 patient.

Q1. Does this patient have his/her PH1 diagnosis confirmed with a genetic test or liver biopsy-based AGT assay?
1= Yes
2= No **[TERMINATE]**

Q2. How was the PH1 diagnosis confirmed for this patient? (Please select all that apply)
1= Genetic testing
2= Liver biopsy-based AGT assay
3= Other clinical assessment/presentation **[TERMINATE]**

Q3. **[Only display Q3 if 1 is selected in Q2]** What AGXT mutations were confirmed by genetic testing? (Please select all that apply)

Mutation in Copy 1	Mutation in Copy 2
1= [Arg36Cys	1= Arg36Cys
2= Asp201Glu	2= Asp201Glu
3= Gly170Arg	3= Gly170Arg
4= Ile244Thr	4= Ile244Thr
5= Met195Arg	5= Met195Arg
6= Phe152Ile	6= Phe152Ile
7= Ser81Leu	7= Ser81Leu
8= Other missense mutation	8= Other missense mutation
9= Other null mutation	9= Other null mutation
10= Unknown mutation	10= Unknown mutation
11= No mutation	11= No mutation

[TERMINATE if 11, "No mutation," is selected for mutation copy 1 OR mutation copy 2]

The dates you enter will not be seen by anyone other than you; the dates will be transposed to different dates once entered and will not disclose the patient's identity.

Q4. When was the patient's diagnosis of PH1 first confirmed with a genetic test or liver biopsy-based AGT assay? (Please use your best approximation.)
_____/_____/_____ (mm/dd/yyyy)

1= Unknown or not available date of diagnosis confirmation with genetic testing or liver biopsy-based AGT assay **[TERMINATE if date value is later than survey_date]**

1. Please document the date of the patient's **first** PH1-related visit **on or after [survey_date - 1096 days]**.
   This date will be referred to as the **index date**.
   _____/_____/_____ (mm/dd/yyyy)
   [TERMINATE if the date is later than [survey_date-1] **or before [survey_date - 1096 days]]**

2. Please document the date of the patient's **last** PH1-related visit.
   _____/_____/_____ (mm/dd/yyyy)

Q5. Does the patient's chart/medical record have information available on the following after the index date on [**first_visit_dt**]?

	**Availability**
PH1-related healthcare resource use (e.g., number of hospitalizations) **[TERMINATE if 2 is selected]**	1 = Yes<br>2 = No
PH1-related signs, symptoms, or clinical manifestations **[TERMINATE if 2 is selected]**	1 = Yes<br>2 = No
PH1-related procedures or treatments	1 = Yes<br>2 = No

[After termination, show the following: If [S7] is greater than the number of charts submitted so far please show the following:]

Q6. Unfortunately, the entered data indicate that the patient selected does not meet the inclusion criteria for this study. Please select an option below to proceed.
1= I have other potentially eligible patients and would like to proceed to identify another patient and verify his/her chart/medical record eligibility for inclusion in this study. **[Please proceed to the beginning Part II]**
2= I do not have other potentially eligible patients, and I would like to exit the survey. **[Exit]**

Part IV – Patient Chart Data Abstraction

PH1 Patient Demographics

The dates you enter will not be seen by anyone other than you; the dates will be transposed to different dates once entered and will not disclose the patient's identity.

Q7. What was the patient's month and year of birth?
_____/_____ (mm/yyyy)
1= Unknown

Q8. What was the patient's gender?
1= Male
2= Female
3= Unknown

Q9. What was the patient's race?
1= White or Caucasian
2= Black
3= Asian
4= Other
5= Unknown

[Display before Q10 and Q11 and on the same screen as Q10 and Q11]
Please complete the following question using information from the <u>index date</u> on [first_visit_dt]. If the information was not collected at that visit, please use the information from the closest visit prior to [first_visit_dt].

[Only display Q10 for US participants]
Q10. What was the primary type of insurance (i.e., disregarding supplemental insurance) the patient had on the index date on **[first_visit_dt]?**
1= Commercial/private insurance
2= Medicare Fee-for-Service
3= Managed Medicare
4= Medicaid Fee-for-Service
5= Managed Medicaid
6= Other public insurance
7= No insurance
8= Unknown

[Only display Q11 for Canadian participants]
Q11. What was the primary type of prescription drug coverage the patient had on the index date on **[first_visit_dt]?**
1= Commercial/private insurance
2= Federal public drug benefit program
3= Provincial or territorial public drug benefit program
4= Other
5= No insurance
6= Unknown

Q12. Is the patient currently alive as of **[survey_date]?**
1= Yes
2= No
3= Unknown

[Only display Q13 if 2=No is selected in Q12]
Q13. What was the patient's age at death?
____ year(s)
1= Unknown

PH1 Clinical Characteristics at Diagnosis

[Repeat this instruction before Q14 to Q17, and show the instruction and each question on the same page] Please complete the following questions using information prior to or at diagnosis of PH1 on

[Q4_dx_dt], where diagnosis is defined as having a confirmatory genetic test or liver biopsy-based AGT assay.

Q14. Did the patient present with any signs and/or symptoms of PH1 prior to or at diagnosis on **[Q4_dx_dt]?**

1= Yes
2= No, the patient did not present with any signs or symptoms
3= Unknown

Sign/symptoms may include but are not limited to:

- Failure to thrive
- Pain (e.g., abdominal/ flank pain, painful urination)
- Fatigue/weakness
- Hematuria (blood in urine)
- Decreased urine output
- Urinary tract infections/pyelonephritis
- Urolithiasis or nephrolithiasis episodes (episodes involving clinically evident urinary tract stones or kidney stones)
- Nephrocalcinosis (too much calcium deposited in kidneys)
- Signs of systemic oxalosis (e.g., arrhythmia, cardiomyopathy, fractures, peripheral neuropathy, respiratory distress, etc.)
- Albuminuria
- Acute renal decline/acute kidney injury (acute eGFR (estimated glomerular filtration rate) decline by 20% or more)
- CKD (chronic kidney disease) or ESRD (end stage renal disease)

[Display Q15 and Q16 if 1 is selected for Q14]

Q15. What presenting signs and/or symptoms did the patient exhibit prior to or at diagnosis on **[Q4_dx_dt]**? (Please select all that apply)

1= Failure to thrive
2= Pain (e.g., abdominal/ flank pain, painful urination)
3= Fatigue/weakness
4= Hematuria (blood in urine)
5= Decreased urine output
6= Urinary tract infections/pyelonephritis
7= Urolithiasis or nephrolithiasis episodes (episodes involving clinically evident urinary tract stones or kidney stones)
8= Nephrocalcinosis (too much calcium deposited in kidneys)
9= Signs of systemic oxalosis (e.g., arrhythmia, cardiomyopathy, fractures, peripheral neuropathy, respiratory distress, etc.)
10= Albuminuria
11= Acute renal decline/acute kidney injury (acute eGFR decline by 20% or more)
12= CKD (chronic kidney disease) or ESRD (end stage renal disease)
13= Other
14= Unknown

Q16. What was the patient's age at onset of the first PH1 sign or symptom that arose prior to or at diagnosis on **[Q4_dx_dt]**? (Please use your best approximation.)

____ years

1= Age at symptom onset was not recorded

[Only display Q17 if 9= signs of systemic oxalosis is selected in Q15]

Q17. What were the specific signs and/or symptoms of systemic oxalosis that were observed prior to or at diagnosis on **[Q4_dx_dt]**? (Please select all that apply)

1= Anemia
2= Arrhythmia
3= Bone pain
4= Cardiomyopathy
5= Cerebral infarction
6= Dental pain
7= Fractures
8= Peripheral neuropathy
9= Refractory hypotension
10= Respiratory distress
11= Skin ulcers
12= Vision problem
13= Other
14= Unknown

Q18. What was the eGFR at diagnosis on **[Q4_dx_dt]?**

______mL/min/1.73m^2

1= eGFR not measured on date of initial diagnostic confirmation of PH1
2= Unknown

PH1-Related Resource Utilization Outcomes Prior to or on the Index Date

The following set of questions will ask for information on the patient's healthcare resource utilization prior to or on the index date on [first_visit_dt].

Q19. Did the patient have any PH1-related hospitalizations prior to or on the index date on **[first_visit_dt]**?

1= Yes
2= No
3= Unknown

Q20. Did the patient have any PH1-related urgent or emergency care visit(s) prior to or on the index date on **[first_visit_dt]**? Please include urgent and emergency care visits that resulted in a hospitalization.

1= Yes
2= No
3= Unknown

PH1-Related Resource Utilization Outcomes After the Index Date

The following set of questions will ask for information on the patient's healthcare resource utilization over the period after the index date from [first_visit_dt + 1 day] to the most recent visit on [last_visit_dt].

Q21. After the index date on **[first_visit_dt]**, how many times did the patient have the types of healthcare-related encounters listed in the table below? For each form of resource use below, please report the total number of PH1-related encounters.

	**Number of PH1-related resource use encounters from [first_visit_dt + 1 day] to [last_visit_dt]**
Hospitalization(s)	_________ **hospitalization(s)**<br>1= Unknown
Length of stay for each hospitalization<br>*Please enter for each hospitalization separately*	**[Only display if [Q21_ip] >0]**<br>**Repeat N=[Q21_ip] times for entry of the length of stay for each hospitalization**<br>Hospitalization 1_________ day(s)<br>1= Unknown<br>Hospitalization 2_________ day(s)<br>1= Unknown
Urgent and emergency care visit(s)<br>*Please include urgent and emergency care visits that resulted in a hospitalization. These visits should be counted as both an emergency department visit and a hospitalization*	_________ urgent and emergency care visit(s)<br>1= Unknown
Outpatient visit(s)	_________outpatient visit(s)<br>1= Unknown
Laboratory test(s)<br>☐ This information was not recorded<br>**[If selected, deactivate this row]**	Number of 24-hour urine oxalate tests: _________ test(s)<br>☐Unknown<br>Number of spot urine oxalate: creatinine ratio tests:<br>_________ test(s)<br>☐Unknown<br>Number of plasma oxalate tests: _________ test(s)<br>☐Unknown<br>Number of serum creatinine tests:_________ test(s)<br>☐Unknown
Renal ultrasound(s)	________ ultrasound(s)<br>☐Unknown
Bone X-ray examination(s)	_________ examination(s)<br>☐Unknown
Electrocardiogram(s) (EKGs)	_________ EKG(s)<br>☐Unknown
Echocardiogram(s)	_________ echocardiogram(s)<br>☐Unknown

PH1 Clinical Outcomes Prior to or on the Index Date

The following set of questions will ask information on the patient's signs, symptoms, and clinical manifestations prior to or on the index date on [first_visit_dt].

Q22. What signs were ever observed in the patient prior to or on the index date on **[first_visit_dt]?** (Please select all that apply)

1= Failure to thrive
2= Hematuria (blood in urine)
3= Decreased urine output
4= Other
5= No signs
6= Unknown

Q23. What symptoms were ever observed in the patient prior to or on the index date on **[first_visit_dt]?** (Please select all that apply)

1= Pain (e.g., abdominal/ flank pain, painful urination)
2= Fatigue/weakness
3= Other
4= No symptoms
5= Unknown

Q24. What other clinical manifestations were ever observed in the patient prior to or on the index date on **[first_visit_dt]?** (Please select all that apply)

1= Urinary tract infections / pyelonephritis
2= Urolithiasis or nephrolithiasis episodes (episodes involving clinically evident urinary tract stones or kidney stones)
3= Nephrocalcinosis (too much calcium deposited in kidneys)
4= Acute renal decline/acute kidney injury (acute eGFR decline by 20% or more)
5= Other
6= No other manifestations
7= Unknown

Q25. Were signs of systemic oxalosis (e.g., arrhythmia, cardiomyopathy, fractures, peripheral neuropathy, respiratory distress, etc.) ever observed in the patient prior to or on the index date on **[first_visit_dt]?**

1= Yes
2= No
3= Unknown

[Only display Q26 if 1 is selected in Q25]

Q26. What were the specific signs of systemic oxalosis that the patient experienced prior to or on index date on **[first_visit_dt]?** (Please select all that apply)

1= Anemia
2= Arrhythmia
3= Bone pain
4= Cardiomyopathy
5= Cerebral infarction
6= Dental pain
7= Fractures
8= Peripheral neuropathy
9= Refractory hypotension
10= Respiratory distress
11= Skin ulcers

12= Vision problem
13= Other
14= Unknown

PH1 Clinical Outcomes After the Index Date

The following set of questions will ask information on the patient's signs, symptoms, and clinical manifestations after the index date, from [first_visit_dt + 1 day] to the most recent visit on [last_visit_dt].

Q27. What signs were ever observed in the patient after the index date, from **[first_visit_dt + 1 day]** to **[last_visit_dt]**? (Please select all that apply)
1= Failure to thrive
2= Hematuria (blood in urine)
3= Decreased urine output
4= Other
5= No signs
6= Unknown

Q28. What symptoms were ever observed in the patient after the index date, from **[first_visit_dt + 1 day]** to **[last_visit_dt]?** (Please select all that apply)
1= Pain (e.g., abdominal/ flank pain, painful urination)
2= Fatigue/weakness
3= Other
4= No symptoms
5= Unknown

Q29. What other clinical manifestations were ever observed in the patient after the index date, from **[first_visit_dt + 1 day]** to **[last_visit_dt]**? (Please select all that apply)
1= Urinary tract infections / pyelonephritis
2= Urolithiasis or nephrolithiasis episodes (episodes involving clinically evident urinary tract stones or kidney stones)
3= Nephrocalcinosis (too much calcium deposited in kidneys)
4= Acute renal decline/acute kidney injury (acute eGFR decline by 20% or more)
5= Other
6= No other manifestations
7= Unknown

[Only display Q30 if 1 or 2 is selected in Q29]
Q30. How many separate times did the patient experience the following clinical events after the index date, from **[first_visit_dt + 1 day]** to **[last_visit_dt]?**

**Clinical event**	**Number of separate occurrences**
[Only display if 1 is selected in Q29]<br>Urinary tract infections / pyelonephritis	_____ occurrence(s)<br>1= Unknown
[Only display if 2 is selected in Q29]<br>Urolithiasis or nephrolithiasis episodes (episodes involving clinically evident urinary tract stones or kidney stones)	_____ stone event(s)<br>1= Unknown

[Only display Q31 if 4 is selected in Q29]

Q31. How many episodes of acute renal decline/acute kidney injury (acute eGFR decline by 20% or more) did the patient have after the index date, from **[first_visit_dt + 1 day]** to **[last_visit_dt]?**

______ episode(s)

1= Unknown

[Only display Q32 if ≥1 is entered in [Q31_re_dcl]]

Q32. For the **[Q31_re_dcl]** episodes of acute renal decline/acute kidney injury that the patient had after the index date, from **[first_visit_dt + 1 day]** to **[last_visit_dt]**, what was the reason for onset and did any episodes result in a permanent loss of kidney function?

[In the table below include [Q31_re_dcl] rows for episodes]

Acute renal decline/acute kidney injury episode	Reason for onset of the episode (please select all that apply)	Did the episode result in a permanent loss of kidney function?	eGFR at onset	eGFR after the episode
**Episode 1 [Include rows until get to [Q31_re_dcl] episodes]**	1= Dehydration<br>2= Effect of concomitant medication<br>3= Infection<br>4= Obstructive stone event<br>5= Nephrocalcinosis<br>6= Other, please specify_____<br>7= Unknown	1= Yes<br>2= No<br>3= Unknown	**[Only display if "Yes" is selected in Q32_perm]**<br>_____.__mL/min/1.73m^2<br>1= Unknown	**[Only display if "Yes" is selected in Q32_perm]**<br>_____.__mL/min/1.73m^2<br>1= Unknown

Q33. Were signs of systemic oxalosis (e.g., arrhythmia, cardiomyopathy, fractures, peripheral neuropathy, respiratory distress, etc.) ever observed in the patient after the index date, from **[first_visit_dt +1 day]** to **[last_visit_dt]?**

1= Yes
2= No
3= Unknown

[Only display Q34 if 1 is selected in Q33]

Q34. What were the specific signs of systemic oxalosis that the patient experienced after the index date, from **[first_visit_dt + 1 day]** to **[last_visit_dt]**? (Please select all that apply)

1= Anemia
2= Arrhythmia
3= Bone pain
4= Cardiomyopathy
5= Cerebral infarction
6= Dental pain
7= Fractures

8= Peripheral neuropathy
9= Refractory hypotension
10= Respiratory distress
11= Skin ulcers
12= Vision problem
13= Other
14= Unknown

[Only display Q35 if 5 or 7 or 9 or 10 is selected in Q34]
Q35. For each of the following manifestations of systemic oxalosis, how many episodes did the patient have after the index date, from **[first_visit_dt + 1 day]** to **[last_visit_dt]?**

Signs of systemic oxalosis	Number of separate occurrences
**[Only display if 5 is selected in Q34]**<br>Cerebral infarction	_____ occurrence(s)<br>1= Unknown
**[Only display if 7 is selected in Q34]**<br>Fractures	_____ occurrence(s)<br>1= Unknown
**[Only display if 9 is selected in Q34]**<br>Refractory hypotension	_____ occurrence(s)<br>1= Unknown
**[Only display if 10 is selected in Q34]**<br>Respiratory distress	_____ occurrence(s)<br>1= Unknown

Patient's Full Lifetime of PH1 Clinical Outcomes

Please complete the following questions with information from the patient's historical and current records in the chart/medical record, regardless of the visit date. Please provide the information to the best of your knowledge.

Q36. Was the patient ever diagnosed with end stage renal disease (ESRD)?
1= Yes
2= No
3= Unknown

The dates you enter will not be seen by anyone other than you; the dates will be transposed to different dates once entered and will not disclose the patient's identity.
[Only display Q37 if 1 is selected in Q36]
Q37. What was the date on which ESRD was first observed?

__/___/_____ (mm/dd/yyyy)
1= Unknown

PH1 Management Characteristics Prior to or on the Index Date

The following set of questions will ask for information on the patient's PH1 management prior to or on the index date on [first_visit_dt].

Q38. Did the patient ever have his/her PH1 managed by any of the following prior to or on the index date on **[first_visit_dt]**? (Please select all that apply)
1= Hyperhydration (drinking large amounts of fluid)

2= Crystallization inhibitors (e.g., citrate supplementation, pyrophosphates, sodium or potassium citrate or pyrophosphate-containing solutions)
3= Pyridoxine supplement
4= None of the above
5= Unknown

Q39. Did the patient ever have any of the following procedures prior to or on the index date on **[first_visit_dt]**? (Please select all that apply)
1= Lithotripsy
2= Ureteroscopy
3= Percutaneous nephrolithotomy
4= Nasogastric tube (NG-tube) placement
5= Gastrostomy tube (G-tube) placement
6= None of the above
7= Unknown

PH1 Management Characteristics After the Index Date

The following set of questions will ask for information on the patient's PH1 management after the index date, from [first_visit_dt + 1 day] to the most recent visit on [last_visit_dt].

Q40. Did the patient ever have his/her PH1 managed by any of the following treatments after the index date, from **[first_visit_dt +1 day]** to **[last_visit_dt]**? (Please select all that apply)
1= Hyperhydration (drinking large amounts of fluid)
2= Crystallization inhibitors (e.g., citrate supplementation, pyrophosphates, sodium or potassium citrate or pyrophosphate-containing solutions)
3= Pyridoxine supplement
4= None of the above
5= Unknown

Q41. Did the patient ever have any of the following procedures after the index date, from **[first_visit_dt +1 day]** to **[last_visit_dt]**? (Please select all that apply)
1= Lithotripsy
2= Ureteroscopy
3= Percutaneous nephrolithotomy
4= Nasogastric tube (NG-tube) placement
5= Gastrostomy tube (G-tube) placement
6= None of the above
7= Unknown

[Only display Q42 if 1 or 2 or 3 is selected in Q41]
Q42. How many stone treatment procedures did the patient after the index date, from **[first_visit_dt +1 day]** to **[last_visit_dt]?**

Procedure	Number of procedures	Adverse events ever associated with any procedures
**[Only display if 1 is selected in Q41]**<br>Lithotripsy	_____ procedure(s)<br>1= Unknown	**[only show if Q42_lithotripsy>0]**<br>1= Bleeding

		2= Urinary tract infections<br>3= Sepsis<br>4= Other infections<br>5= Ureteral stricture<br>6= Ureteral injury<br>7= Ureteral steinstrasse<br>8= Other, please specify___________<br>9= No AEs were reported<br>10= Unknown if AE occurred
**[Only display if 2 is selected in Q41]**<br>Ureteroscopy	_____ procedure(s)<br>1= Unknown	**[only show if Q42_ ureteroscopy >0]**<br>1= Bleeding<br>2= Urinary tract infections<br>3= Sepsis<br>4= Other infections<br>5= Ureteral stricture<br>6= Ureteral injury<br>7= Ureteral steinstrasse<br>8= Other, please specify ___________<br>9= No AEs were reported<br>10= Unknown if AE occurred
**[Only display if 3 is selected in Q41]**<br>Percutaneous nephrolithotomy	_____ procedure(s)<br>1= Unknown	**[only show if Q42_ nephrolithotomy >0]**<br>1= Bleeding<br>2= Sepsis<br>3= Other infection<br>4= Urinoma<br>5= Thoracic complications<br>6= Injury to surrounding organ (e.g., bowel, spleen, liver)<br>7= Death<br>8= Other, please specify_______<br>9= No AEs were reported<br>10= Unknown if AE occurred

[Only display Q43 if 4 is selected in Q39 OR Q41]

Q43. Did the patient ever have any of the following adverse events associated with a nasogastric tube (NG-tube) placement? (Please select all that apply)

1= Aspiration
2= Esophageal perforation
3= Nostril erosion
4= Pneumothorax
5= Sinusitis
6= Other, please specify ______________
7= No AEs were reported

8= Unknown if AE occurred

[Only display Q44 if 5 is selected in Q39 OR Q41]

Q44. Did the patient ever have any of the following adverse events associated with a gastrostomy tube (G-tube) placement? (Please select all that apply)

1= Bleeding
2= Bowel obstruction
3= Sepsis
4= Other infections
5= Stomal leakage
6= Tube dislodgement or migration
7= Bowel perforation
8= Other, please specify ______________
9= No AEs were reported
10= Unknown if AE occurred

Patient's Full Lifetime of PH1 Management Characteristics

The following set of questions will ask information on the patient's full lifetime of PH1 management, including all historical and current management, please provide all information to the best of your knowledge.

Q45. Did the patient ever have dialysis?

1= Yes
2= No
3= Unknown

[Only display Q46 if 1 is selected in Q45]

Q46. What is the initiation date, date of most recent dialysis, and regimen of the most recent dialysis procedure the patient had?

**Date of dialysis initiation**	**Date of most recent dialysis**	**Most recent dialysis type and regimen**	**Adverse events associated with any dialysis procedures**
**Date:**<br>____/_____/____<br>**(mm/dd/yyyy)**<br>1= Unknown	**Date:**<br>____/_____/_____<br>**(mm/dd/yyyy)**<br>1= Unknown	Dialysis type:<br>**Please insert a drop down menu:**<br>• **Peritoneal dialysis**<br>• **Hemodialysis**<br>• **Both**<br><br>**[If "Hemodialysis" or "Both" was selected in the drop down, ask regimen]**<br>Hemodialysis regimen	**(Please select all that apply)**<br>1= Sepsis<br>2= Other infections<br>3= Access site complications<br>4= Muscle cramps<br>5= Bone/joint pain<br>6= Itchy skin<br>7= Hypotension<br>8= Nausea/vomiting<br>9= Insomnia<br>10= Anxiety or depression<br>11= Fatigue<br>12= Hernia

		------times per week (please enter a whole number):<br><br>-----hours per session(please enter a number in intervals of 0.5)	13= Other, please specify___________<br>14= No AEs were reported<br>15= Unknown if AE occurred

Q47. Did the patient ever have any of the following transplant procedures? (Please select all that apply)
1= Isolated kidney transplant
2= Isolated liver transplant
3= Combined liver-kidney transplant
4= None of the above
5= Unknown

[Only display Q48 if 4 or 5 is NOT selected in Q47]
Q48. When did the patient have the transplant procedure?

**Procedure**	**Number of procedures**	**Age at time of procedures**	**Adverse events associated with any transplant procedures (Please select all that apply)**
**[Only display if 1 is selected in Q47]**<br>Isolated kidney transplant	**_____ procedure(s)**<br>1= Unknown	**____ year(s)**<br>1= Unknown<br><br>**[Repeat age field Q48_kt times]**	**[only show if Q48_kt >0]**<br>1= Failure of donated kidney<br>2= Rejection of donated kidney<br>3= Bleeding<br>4= Blood clots<br>5= Sepsis<br>6= Other infections<br>7= Cancer<br>8= Heart attack<br>9= Stroke<br>10= Confusion<br>11= Seizure<br>12= Urine leak<br>13= Death<br>14= Other, please specify___________<br>15= No AEs were reported<br>16= Unknown if AE occurred
**[Only display if 2 is selected in Q47]**	**_____ procedure(s)**<br>1= Unknown	**____ year(s)**<br>1= Unknown	**[only show if Q48_lt >0]**

Isolated liver transplant		**[Repeat age field Q48_lt times]**	1= Failure of donated liver<br>2= Rejection of donated liver<br>3= Bleeding<br>4= Blood clots<br>5= Sepsis<br>6= Other infection<br>7= Cancer<br>8= Heart attack<br>9= Stroke<br>10= Confusion<br>11= Seizure<br>12= Bile duct leak<br>13= Bile duct stricture<br>14= Death<br>15= Other, please specify___________<br>16= No AEs were reported<br>17= Unknown if AE occurred
**[Only display if 3 is selected in Q47]**<br>Combined liver-kidney transplant	_____ **procedure(s)**<br>1= Unknown	____ **year(s)**<br>1= Unknown<br><br>**[Repeat age field Q48_lk times]**	**[only show if Q48_lk >0]**<br>1= Failure of donated kidney and/or liver, please specify__________<br>2= Rejection of donated kidney and/or liver, please specify___________<br>3= Bleeding<br>4= Blood clots<br>5= Sepsis<br>6= Other infection<br>7= Cancer<br>8= Heart attack<br>9= Stroke<br>10= Confusion<br>11= Seizure<br>12= Urine leak<br>13= Bile duct leak<br>14= Bile duct stricture<br>15= Death<br>16= Other, please specify___________<br>17= No AEs were reported

			18= Unknown if AE occurred

Laboratory Tests

The following set of questions will ask for information on the patient's PH1 laboratory tests during the 1 month prior to or on the index date on [first_visit_dt].

Q49. Please provide lab value on the index date on **[first_visit_dt]**. If no labs were taken on the <u>index date</u>, please provide a lab value that occurred between **[first_visit_dt - 31]** and **[first_visit_dt]** if available

	**Closest measure on or prior to the index date on [first_visit_dt]**	
**Lab measure**	**Lab value (Please keep only one decimal)**	**Lab unit [Only show if a value is entered for the lab value]**
24-hour urine oxalate	_____.__<br>1= Unknown<br>2= No 24-hour urine oxalate lab in the 1 month prior to or on **[first_visit_dt]**	1= mmol / 1.73m^2 / day<br>2= mg / 1.73m^2 / day<br>3= mmol / day<br>4= mg / day<br>5= Other, please specify _____<br>6= Unknown
Spot urine oxalate: creatinine ratio	_____.__<br>1= Unknown<br>2= No spot urine oxalate: creatinine ratio labs in the 1 month prior to or on **[first_visit_dt]**	1= mmol/mol<br>2= mmol/mmol<br>3= mg/mg<br>4= Other, please specify _____<br>5= Unknown
Plasma oxalate	_____.__<br>1= Unknown<br>2= No plasma oxalate lab in the 1 month prior to or on **[first_visit_dt]**	1= mcmol/L or µmol/L<br>2= Other, please specify _____<br>3= Unknown
eGFR	_____.__<br>1= Unknown<br>2= No eGFR lab in the 1 month prior to or on **[first_visit_dt]**	1= mL/min/1.73m^2<br>2= Other, please specify _____<br>3= Unknown

Thank you for recording the information about this patient. Your answers for this patient have been successfully recorded. You have submitted information on **<pipe in number of cases> patient(s)**. If you have additional eligible patients, please select "Yes" below to add them now.

Would you like to add another patient record now or at a later time?

Yes – Go back to start of PART II
No – End Survey

Please note, if you select Yes above, but then pause the survey, you will need to resume this survey, complete the patient record you started, and then select "No - End Survey" when you get back to this question again in order to submit your responses and be counted as a complete to receive your incentive. If you pause the survey and do not make your way back to this question to select "No – End Survey", your responses will not be recorded, and you will not be counted as a complete to receive your incentive.

Thank you for your participation in this study.

Supplemental Figure 2. Incidence rate of acute events associated with PH1 during follow-up

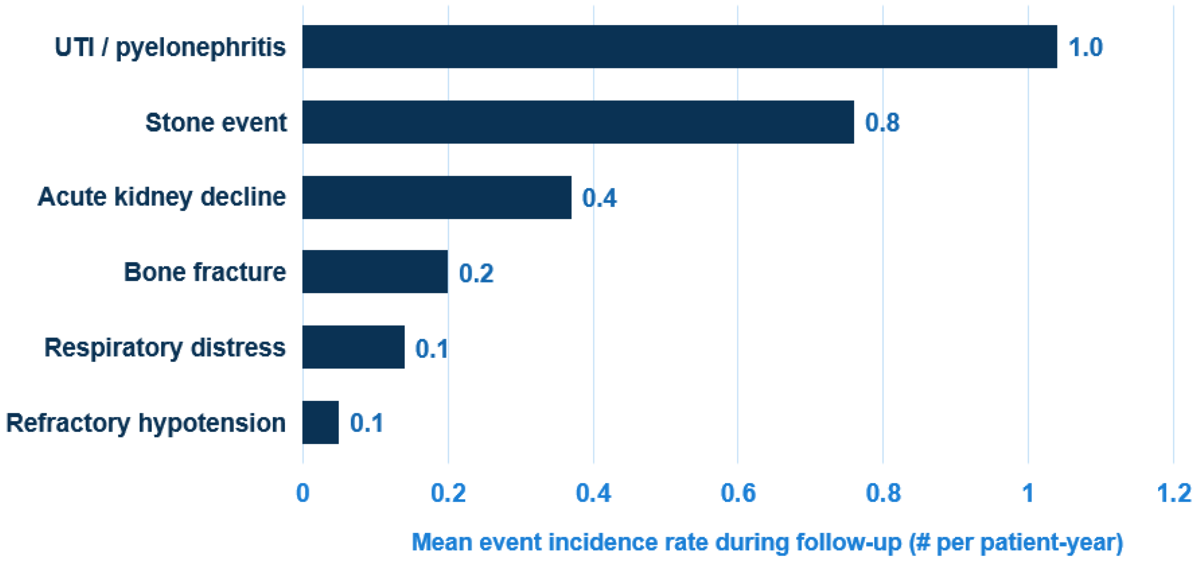

Abbreviation: UTI: urinary tract infection.
Note: Data are reported among all patients with a known number of events. Acute kidney decline was defined as an acute eGFR decline by 20% or more.

Supplemental Figure 3. Reasons for onset of acute kidney decline in PH1

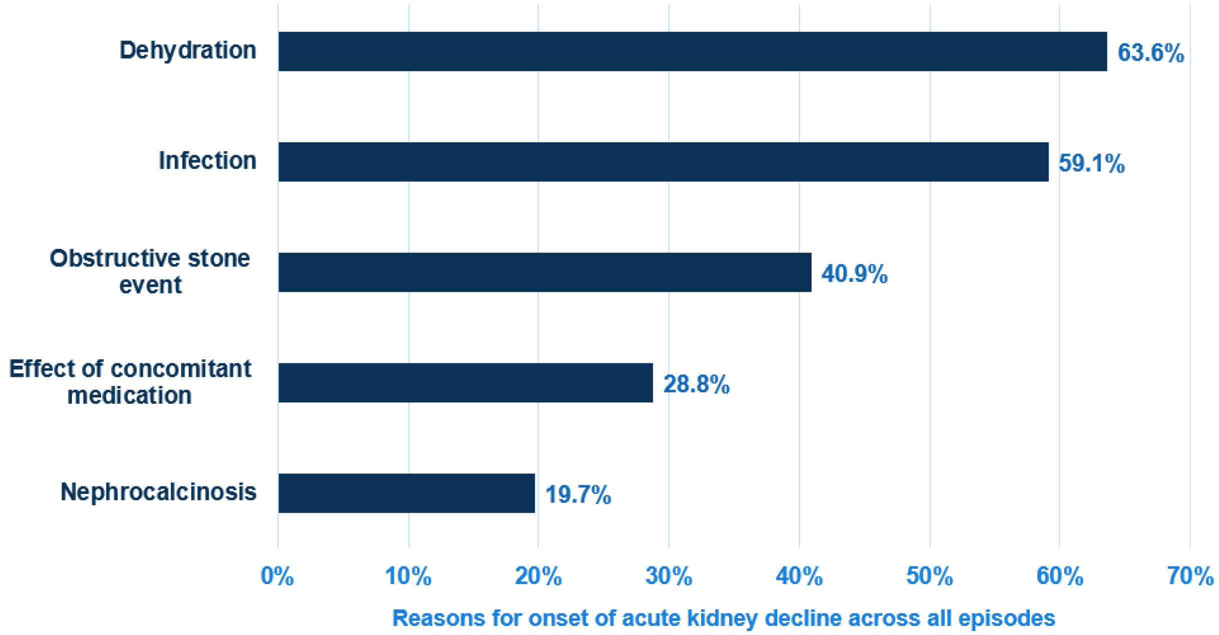

Supplemental Figure 4. Prevalence of extrarenal manifestations of oxalosis during follow-up

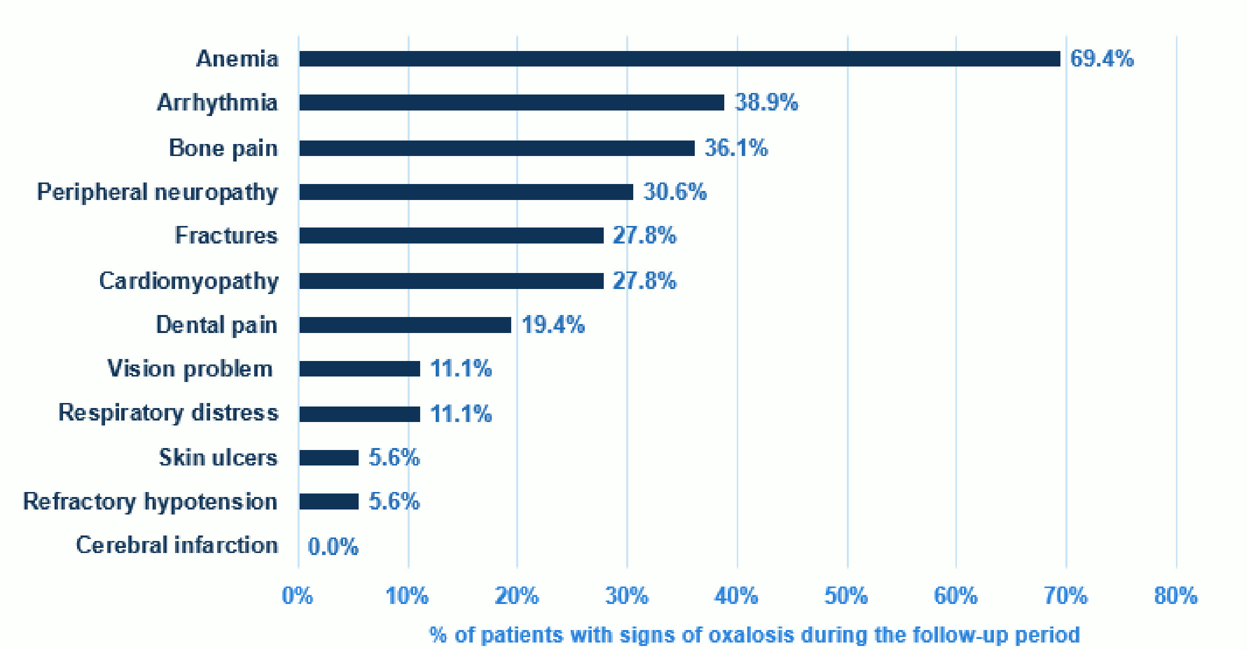

Note: Data are reported among 36 patients with known extrarenal manifestations of oxalosis.
